# Association of TORCH Pathogens With Spontaneous Pregnancy Loss: A Prospective Study From Pakistan

**DOI:** 10.1002/mbo3.70075

**Published:** 2025-10-28

**Authors:** Akmal Zubair, Ahmed Al‐Emam, Hesham M. Hassan, Luigi Santacroce, Ranya Mohammed Elmagzoub

**Affiliations:** ^1^ Department of Biotechnology Quaid‐i‐Azam University Islamabad Pakistan; ^2^ Department of Pathology College of Medicine King Khalid University Asir Saudi Arabia; ^3^ Department of Pathology College of Medicine King Khalid University Abha Saudi Arabia; ^4^ Interdisciplinary Department of Medicine, Section of Microbiology and Virology School of Medicine University of Bari Bari Italy; ^5^ Department of Biology and Biotechnology Faculty of Science and Technology Al‐Neelain University Khartoum Sudan

**Keywords:** abortion, fetus, IgG, IgM, Pakistan, pregnant, TORCH agent

## Abstract

This study aimed to determine the prevalence of TORCH infections (*Toxoplasma gondii*, Rubella virus, Cytomegalovirus [CMV], and herpes simplex virus [HSV]) among pregnant women reported at Pakistan Institute of Medical Sciences (PIMS) Hospital in Pakistan. The study was conducted at PIMS from May 2021 to September 2022. This prospective study analyzed 8000 samples submitted to the pathology laboratory for routine testing. The ELISA method was employed to detect immunoglobulin M and immunoglobulin G antibodies. Statistical analysis was conducted using SPSS (version 23.0). TORCH agent infections during pregnancy account for 19.82% of cases. *T. gondii* is responsible for 5.1% of complete abortions, 6.6% of incomplete abortions, 4.8% of missed abortions, and 2.8% of threatened abortions. Rubella is another pathogen that can lead to abortion in pregnant women, contributing to 7.8% of complete abortions, 19.7% of incomplete abortions, 5.7% of missed abortions, and 5.5% of threatened abortions. CMV and HSV are also implicated in abortion. CMV accounts for 6.2% of complete abortions, 9.1% of incomplete abortions, 4.8% of missed abortions, and 5.2% of threatened abortions. HSV plays a significant role in abortion as well, causing 4.2% of complete abortions, 7.1% of incomplete abortions, 1.5% of missed abortions, and 4.0% of threatened abortions. Data with a *p* value of less than 0.001 indicate strong statistical significance. The findings suggest that TORCH agents and abortion are significantly correlated, with TORCH agent infections being a major cause of abortion.

## Graphical abstract

Women aged 15–44 had 50 abortions per 1000 in poor nations like Pakistan. Religion keeps abortion‐related issues like TORCH agent infection quiet in Pakistan. This is the first detailed report of TORCH infection in Pakistan. *Toxoplasma gondii*, Rubella, Cytomegalovirus, and herpes simplex virus cause 19.3%, 38.7%, 25.3%, and 16.8% of abortions. Pakistan has an alarming rate of abortions, and immediate action is needed.

## 1 Introduction

Toxoplasmosis, Rubella virus, Cytomegalovirus (CMV), and herpes simplex virus (HSV) infections are collectively referred to as TORCH infections. These infections are a leading cause of serious complications during pregnancy. Often, the severity of the infection poses a greater risk to the fetus than to the mother (Wang et al. 2019; Al‐Hakami et al. 2020). Transplacental transmission allows these pathogens to enter the fetal bloodstream. Furthermore, this transmission can occur at any stage of gestation or, in some cases, just before delivery (Mahmoud et al. 2023; Naseem et al. 2025). Compared to recurrent infections, primary infections have a higher mortality rate and can result in congenital abnormalities, miscarriage, sudden intrauterine death, growth retardation, preterm birth, and live‐born infants who exhibit signs of disease (Baghel and Inamdar 2020). These infectious diseases significantly increase mortality and morbidity rates in developing countries (Zuo et al. 2022). Abortions (10%–15%) are attributed to TORCH infections (Kalantari et al. 2021). These infections are more prevalent in some countries than in others. Specific immunoglobulin M (IgM) antibodies can be used to identify these maternal infections. When a fetus contracts various illnesses in the womb, it can result in multiple symptoms that may affect the child's health at birth. Maternal risk factors include inadequate vaccination, sexually transmitted diseases (STDs), and exposure to animals during pregnancy (Adgoy et al. 2020). These infections are more prevalent in some countries than in others. Specific IgM antibodies can be used to identify these maternal infections. When a fetus is infected with various illnesses in the womb, it can result in multiple symptoms that may affect the child's health at birth. Maternal risk factors include inadequate vaccination, STDs, and exposure to animals during pregnancy (Belanger and Lui 2023). Rubella, *Toxoplasma gondii*, CMV, human immunodeficiency virus (HIV), hepatitis B virus, HSV types 1 and 2 (HSV‐1 and HSV‐2), along with other pathogens, such as syphilis, parvovirus, and varicella, are classified as TORCH infections. However, our primary focus will be on Rubella, *T. gondii*, CMV, and HSV (Abd‐elgawad and Mohamed 2019; Leung 2020). Infections can spread during pregnancy through the transplacental pathway, during delivery via blood, or through vaginal secretions during labor and delivery. Infections that occur after delivery generally have a lesser impact. Certain infections, such as syphilis, hepatitis B, and HIV, can be transmitted through sexual contact with an infected mother (Mahmood and Kahya 2021). Proper immunization of mothers can prevent the spread of varicella and Rubella. Perinatal infections account for 2%–3% of all congenital abnormalities (Manjunathachar et al. 2020). Initial signs of infection may appear during pregnancy, at birth, within the first year of a baby's life, or even years later (Khan and Chatterjee 2019). Congenital infections can affect the fetus during pregnancy, resulting in abnormal growth or developmental patterns. Newborn infants with these infections may exhibit a variety of clinical issues, including growth abnormalities and developmental defects. Many clinical symptoms of viral infections may manifest within the first few days after birth (Irina and Valeriu 2023). They typically cause a rash that may be purpuric, petechial, or maculopapular (often referred to as a sensorineural hearing loss (especially associated with CMV), and chorioretinitis. Hepatosplenomegaly and cardiac abnormalities are also common complications (Liu et al. 2020; Goud et al. 2021). *Toxoplasmosis* and maternal CMV affect 2–10 neonates per 1000 (Goud et al. 2021). Because HSV‐2 primarily causes genital infections, humans are the natural hosts of this herpes virus (Kakayi et al. 2021). Babies frequently contract viruses. Exposure to cats and the consumption of improperly prepared foods, such as undercooked meat or unpasteurized dairy products, are risk factors for toxoplasmosis (Dawood 2019; Chen et al. 2019)

Since few studies have investigated the seroprevalence of TORCH agents at the Pakistan Institute of Medical Sciences (PIMS) in Islamabad, Pakistan, the present study was designed to assess both immunoglobulin G (IgG) and IgM levels in a larger sample size to obtain more significant results. The current situation in Pakistan indicates that TORCH infections contribute substantially to neonatal morbidity and mortality; however, they remain underdiagnosed due to inadequate diagnostic facilities, particularly in rural areas. Pregnant women are rarely tested for TORCH pathogens unless they experience complications, such as recurrent miscarriages or congenital anomalies. Furthermore, the absence of standardized screening programs in antenatal care increases the risk of undetected maternal infections being transmitted to the fetus. Public awareness of TORCH infections is also minimal, leading to delays in seeking medical attention and poor pregnancy outcomes. To address this study gap, Pakistan requires a systematic approach that includes nationwide studies to determine prevalence rates, improved diagnostic infrastructure, and the integration of TORCH screening into routine maternal healthcare programs.

TORCH‐related infections are critical to preventing pregnancy complications, including congenital birth defects, stillbirths, and pregnancy delays. Early diagnosis and detection are essential to avoid long‐term effects. Research on TORCH infections can improve our ability to identify TORCH agents early, develop new protocols, and provide timely guidance and preventive strategies that benefit both the mother and fetus.

## 2 Materials and Methods

### 2.1 Population Study

The prospective study included 1274 pregnant women out of a total of 8000 participants, representing females of all ages, socioeconomic classes, and various regions of Pakistan. The demographic data are presented in Table 1.

**Table 1:** The demographic data of pregnant females.

Risk factor	Variable	Number
Age	20–30	4350
	31–40	2732
	> 40	918
Education level	Uneducated	5320
	Primary education	2200
	Higher diploma	480
Job	House holder	6549
	Job holders	1451
Region	Islamabad	28
	Khyber Pakhtunkhwa	2340
	Punjab	4400
	Sind	79
	Balochistan	566
Family income	< 20,000 PKR	5687
	< 50,000 PKR	1457
	> 50,000 PKR	856
No. of children	None	3600
	One	2311
	Two	2089
Trimester	First	5487
	Second	2513
Adverse pregnancy	Yes	4929
	No	3071

*Note:* The demographic data represent the marital status, age, family income, and so forth.

### 2.2 Pregnancy Check‐Up

All pregnant women underwent a routine checkup, which included measurements of body weight, abdominal circumference, and blood pressure, followed by blood and urine tests.

### 2.3 Sample Collection

A total of 8000 samples were received at the pathology laboratory for diagnostic purposes. The samples were sent to the PIMS Hospital for analysis between May 2021 and September 2022, during which the study was conducted. The demographic data of the samples are presented in Table 1. A simple random sampling method was employed to collect these samples, minimizing potential biases. During the procedure, any female presenting with pregnancy‐related issues was tested for TORCH infections, regardless of age, ethnicity, height, number of children, geographic location, or family size. The investigation included 8000 samples from pregnant women who visited various clinics for routine check‐ups. Eight milliliters of blood were drawn aseptically into a plain tube without anticoagulant. The separated serum was stored at −20°C.

### 2.4 Procedure

**Figure 1:**
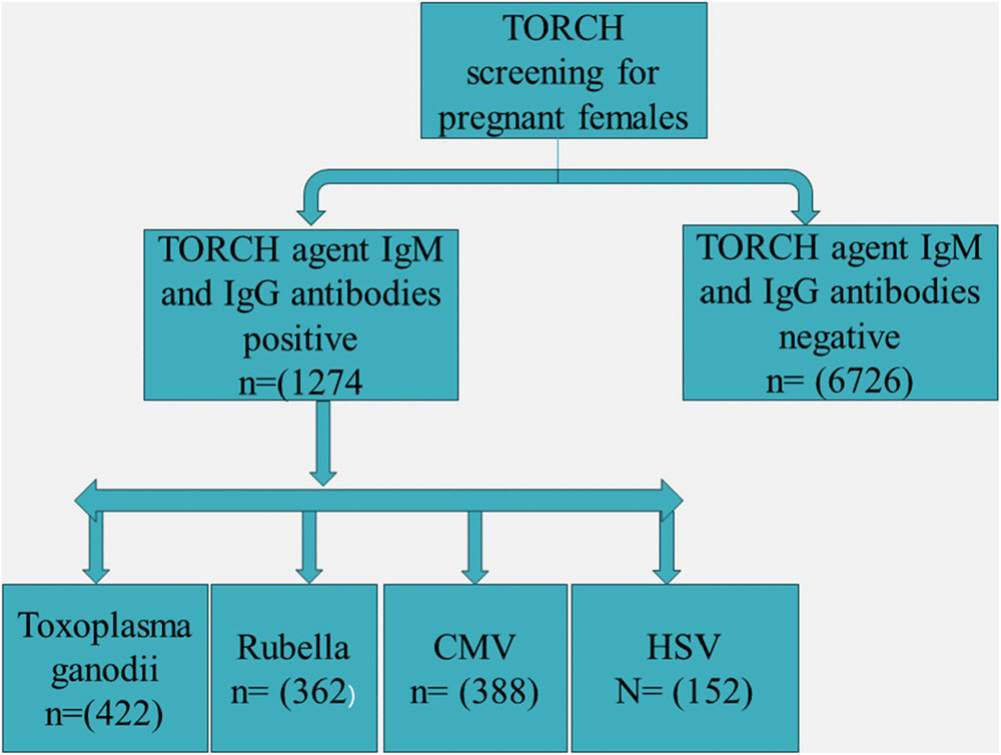
Flowchart of the study. The study represents the number of females screened for various TORCH agents. The TORCH negative females are 6726 and TORCH infected females are 1274. CMV, Cytomegalovirus; HSV, herpes simplex virus; IgG, immunoglobulin G; IgM, immunoglobulin M.

These samples were examined using the enzyme‐linked immunosorbent assay (ELISA) technique in accordance with the manufacturer's instructions (Certification ISO 13485, Model Number TY0027) to determine the presence of IgM and IgG antibodies against Toxoplasma, CMV, Rubella, and Herpes simplex. Before testing the samples, both positive and negative controls were employed. The ELISA method was chosen for this study due to its sensitivity in detecting IgM and IgG antibodies. A sample was deemed positive if the test value exceeded 1.2 and negative if it fell below 1.0. Values ranging from 1.0 to 1.2 were classified as ambiguous. For individuals with borderline sample results, the test was repeated after 1–2 weeks. This investigation included samples from expectant women suspected of having a TORCH infection, while women with a chronic TORCH infection were excluded. Figure 1 illustrates the screening of patients and their respective infection rates.

### 2.5 Inclusion Criteria

We included all pregnant women experiencing complications related to abortion, such as complete abortions, incomplete abortions, missed abortions, and threatened abortions.

### 2.6 Exclusion Criteria

We excluded all females who did not experience abortion‐related complications, as well as those with incomplete information in the hospital records. Induced abortions were also excluded from the study.

### 2.7 Data Analysis

Data were imported into Microsoft Excel (version 2016) for organization and subsequently transferred to SPSS (version 23.0) for Windows 10 program for statistical evaluation analyses. Mean and deviation values were obtained and calculated. Appropriate statistical tests, including the Chi‐square test, were performed. *p* Value of < 0.001 was statistically significant with a 95% interval. Presentations, including pie charts and charts, were created using Microsoft Excel (version 2016).

### 2.8 Ethical Consideration

The ASRB of Quaid‐E‐Azam University, Islamabad, Pakistan approved the current study.

## 3 Results

A total of 8000 blood samples were collected from pregnant women for this study. The ELISA technique was used to measure the concentrations of IgM and IgG antibodies against TORCH pathogens in these samples. The average age of the participants was 24.8 years. Of the 8000 samples, 1274 tested positive for antibodies against toxoplasmosis, CMV, Rubella, and HSV (see Table 2), while 6726 samples tested negative for these infections.

**Table 2:** The concentration of IgG and IgM in a pregnant woman against TORCH.

Antibodies and pathogens cross‐tabulation					
	Pathogen				Total
	Toxoplasma	Rubella	CMV	HSV	
*Antibodies*					
IgG					
Count	155	232	199	125	711
% of total	12.2	18.2	15.6	9.8	55.8
IgM					
Count	267	130	139	27	563
% of total	21.0	10.2	10.9	2.1	44.2
*Total*					
Count	422	362	338	152	1274
% of total	33.1	28.4	26.5	11.9	100.0

*Note:* Pearson Chi‐square value is 116.682*a* and *p* value 0.000. IgG was reported at 55.8%, and IgM at 44.2% against all TORCH infections under study.

Abbreviations: CMV, Cytomegalovirus; HSV, herpes simplex virus; IgG, immunoglobulin G; IgM, immunoglobulin M.

Table 2 presents a cross‐tabulation illustrating the distribution of IgG and IgM antibodies across four pathogens: Toxoplasma, Rubella, CMV, and HSV. The Pearson Chi‐square test yielded a value of 116.682 with a *p* value of < 0.001, indicating a statistically significant association between antibody type (IgG vs. IgM) and pathogen type. This finding suggests that the presence of IgG or IgM is not randomly distributed among these pathogens; rather, certain pathogens are more likely to be associated with one antibody type over the other. For example, IgM, which indicates a recent infection, is more prevalent in cases of Toxoplasma, while IgG, which signifies past infection or immunity, is more frequently observed in cases of Rubella and CMV. The extremely low *p* value further confirms that this pattern is unlikely to have occurred by chance.

**Figure 2:**
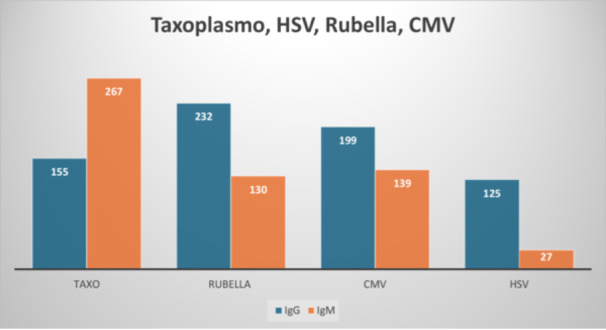
This graph shows pregnant females' IgM and IgG production. CMV, Cytomegalovirus; HSV, herpes simplex virus; IgG, immunoglobulin G; IgM, immunoglobulin M.

The human body responds to foreign antigens, and this immune response is a critical tool in diagnostic studies. In TORCH infections, the body produces IgM and IgG antibodies, with levels varying among women depending on the severity of the infection, as illustrated in Figure 2.

We categorized the infected women based on their age, as shown in Table 3. They were divided into three groups: Group A includes female patients aged 17–30 years, Group B comprises those aged 31–40 years, and Group C consists of individuals over 40 years. Group A, representing younger infected females, exhibited higher levels of IgA and IgM antibodies, while Group C had the lowest levels of IgM and IgG. This suggests that females in Group A have a higher rate of infection, whereas Group C shows the lowest infection levels. Patients in Group A tend to produce more antibodies to combat the infection. The prevalence of infection among females in Group A is 49.5%, compared with 35.3% in Group B and 15.1% in Group C.

**Table 3:** The infection ratio and antibody production at different ages of pregnant females.

Age ∗ Disease cross‐tabulation									
	Disease								
	Taxo IgG	Taxo IgM	Rubella IgG	Rebella IgM	CMV IgG	CMV IgM	HSV IgG	HSV IgM	Total
*Age*									
Group A: 17–30 years									
Count	67	145	98	45	68	96	56	14	589
% of total	5.6	12.2	8.2	3.8	5.7	8.1	4.7	1.2	49.5
Group B: 31–40 years									
Count	65	56	89	65	42	26	68	9	420
% of total	5.5	4.7	7.5	5.5	3.5	2.2	5.7	0.8	35.3
Group C: Above 40 years									
Count	23	66	45	15	9	17	1	4	180
% of total	1.9	5.6	3.8	1.3	0.8	1.4	0.1	0.3	15.1
*Total*									
Count	155	267	232	130	199	139	125	27	1189
% of total	13.0	22.5	19.5	10.20	15.62	11.7	10.5	2.3	100.0

*Note:* Pearson Chi‐square value is 116.035*a* and *p* value 0.000. Group A is the population most affected by TORCH agents, while Group C is the least affected population by TORCH infection.

Abbreviations: CMV, Cytomegalovirus; HSV, herpes simplex virus; IgG, immunoglobulin G; IgM, immunoglobulin M.

**Figure 3:**
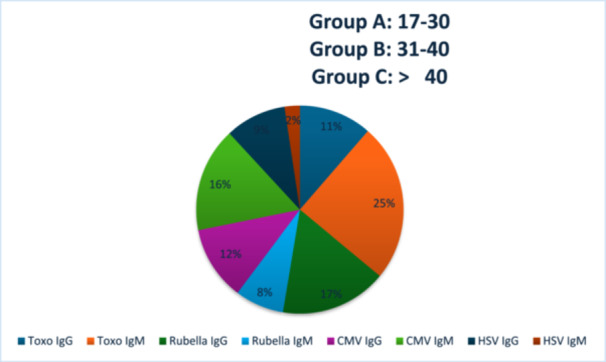
The concentration of IgM and IgG in response to the TORCH agent. HSV IgM shows the highest concentration of all antibodies. CMV, Cytomegalovirus; HSV, herpes simplex virus; IgG, immunoglobulin G; IgM, immunoglobulin M.

Antibody production varies among pregnant females, and its concentration fluctuates with age. We analyzed antibody concentrations according to age groups. All pregnant females were categorized into three groups, as shown in Table 3 and Figure 3: Groups A–C. The immune response to infection was evaluated by measuring the production of IgM and IgG antibodies.

TORCH agents are associated with abortions in pregnant women. We categorized these abortions into four types: complete abortion, incomplete abortion, missed abortion, and threatened abortion, as shown in Table 4. The incidence of these abortion types in relation to *T. gondii*, Rubella, CMV, and HSV is presented in Table 4. The *p* values are less than 0.001, indicating strong evidence of a correlation between TORCH agents and abortions, as well as abortion‐related complications. The overall incidence of TORCH infections during pregnancy is 19.82%. Specifically, *T. gondii* accounts for 5.1% of complete abortions, 6.6% of incomplete abortions, 4.8% of missed abortions, and 2.8% of threatened abortions. Rubella is also implicated in abortions among pregnant women, being responsible for 7.8% of complete abortions, 19.7% of incomplete abortions, 5.7% of missed abortions, and 5.5% of threatened abortions. Additionally, both CMV and HSV contribute to abortion. CMV is responsible for 6.2% of complete abortions, 9.1% of incomplete abortions, 4.8% of missed abortions, and 5.2% of threatened abortions. The presence of HSV is also significant in abortion cases, accounting for 4.2% of complete abortions, 7.1% of incomplete abortions, 1.5% of missed abortions, and 4.0% of threatened abortions. These results indicate that TORCH agents collectively contribute to abortions in pregnant women. However, our findings suggest that Rubella is more prevalent in the samples collected from the Lab of PIMS in Islamabad, Pakistan.

**Table 4:** The abortion, incomplete abortion, missed abortion, and threatened abortion percentages by different TORCH agents.

Pathogen ∗ type of abortion cross‐tabulation					
	Type abortion				
	Complete abortion	Incomplete abortion	Missed abortion	Threatened abortion	Total
*Pathogen*					
Toxo					
Count	81	105	76	44	306
% of total	6.3	8.2	5.9	3.4	24.1
Rubella					
Count	96	102	91	87	376
% of total	7.5	8.0	5.7	5.5	29.5
CMV					
Count	98	104	76	83	361
% of total	7.6	8.1	5.9	6.5	28.3
HSV					
Count	67	77	23	64	231
% of total	5.2	6.1	1.8	5.0	18.1
*Total*					
Count	342	388	266	278	1274
% of total	26.84	30.4	20.8	21.5	100.0

*Note:* Pearson Chi‐square value is 63.068*a* and *p* value 0.000. Table 3 indicates that the most common causative agent of abortions is the Rubella virus, which accounts for 29.5% of all abortion cases.

Abbreviations: CMV, Cytomegalovirus; HSV, herpes simplex virus; IgG, immunoglobulin G; IgM, immunoglobulin M.

## 4 Discussions

### 4.1 Main Findings

Our research indicates that in PIMS Pakistan, the prevalence of *T. gondii*, Rubella, CMV, and HSV among pregnant women was 19.3%, 38.7%, 25.3%, and 16.8%, respectively. The study indicates that Rubella infection has a higher prevalence compared with the other TORCH infections.

### 4.2 Comparison with Previous Studies

Studies conducted in India have revealed a high incidence of TORCH infections, reaching up to 80%, alongside a low prevalence of 5% (Dinçgez Çakmak et al. 2018; Jabbar 2023). In the current investigation, IgM antibodies were detected in 6.7% of 764 samples, while IgG antibodies were present in 58.25% of samples positive for Toxoplasma infection. The prevalence of *T. gondii* infections varies between 7.7% and 76.7% across different countries, with the UK reporting a prevalence of 7.7%–9.11%, and India reporting 45.2% (Manjunathachar et al. 2020), Norway 11.2% (Vilibic‐Cavlek et al. 2011; Nwachukwu 2023) 74.45%, and Brazil 50%–76% (Allain et al. 1998; Tamer et al. 2009). Our findings closely align with those of an Indian study, which reported that 3.47% of participants had IgM antibodies against *T. gondii* (Nwachukwu 2023; Karad and Kharat 2015). The report indicating that 19.4% of patients in India have IgM antibodies specific to Toxoplasma differs from the findings of the current investigation. This discrepancy arises because the previous study included patients with a history of abortion. In our study, we found that 130 samples (23%) tested positive for IgM antibodies against Rubella. In Turkey, de la Galván‐Ramírez (1995) and Warnecke et al. (2020) found that 0.2% of pregnant women tested positive for anti‐Rubella IgM antibodies. CMV, a member of the herpesvirus family, is prevalent worldwide, particularly in areas with low socioeconomic conditions (Numan et al. 2015). The majority of CMV infections remain asymptomatic, which makes clinical diagnosis challenging. In the present study, varying levels of IgM and IgG antibodies against CMV were detected. Additionally, between 17.5% and 52.3% of women who had experienced unplanned pregnancies or abortions tested positive for toxoplasmosis (Dinçgez Çakmak et al. 2018). Age, nutritional status, sociocultural practices, regional climate variations, modes of transmission, and toxoplasmosis seropositivity all have an impact (Adgoy et al. 2020). The infection this parasite causes affects one‐third of the world's population (Jabbar 2023). In the current study, 23% of women with high‐risk pregnancies tested positive for IgG antibodies, indicating a past infection, while 22.3% tested positive for both IgG and IgM antibodies, suggesting a recent infection. In the same region, the prevalence of Toxoplasma seropositivity was 14.6%, based solely on IgM seropositivity and a smaller sample size (Güzel 2020; Sunil and Valsan 2020). Research from the West Zone by Kar et al. (2021), Chattopadhyay (2019), and Manjunathachar et al. (2020) revealed that the seropositivity was 28.5%, 42.10%, and 46.7% for IgG and 9.6%, 10.52%, and 41.3% for IgM, respectively.

### 4.3 Impact of TORCH Infection on Pregnancy

Women with a history of preterm labor exhibited the highest levels of Toxoplasma seropositivity, which was associated with various adverse obstetric outcomes. These included women with a history of intrauterine fetal death, recurrent pregnancy losses, fetal congenital malformations in the current pregnancy, a history of congenital malformations in previous pregnancies, and, to a lesser extent, those with a record of prior neonatal mortality. Despite this (Baghel and Inamdar 2020), a study conducted in the North Zone revealed that among pregnant women with a history of bad obstetric history, the highest seropositivity was observed in cases of abortions (71.8%), followed by preterm deliveries (26.9%), stillbirths (22.2%), miscarriages (11.9%) (Turbadkar et al. 2003; Rajani 2018), congenital abnormalities (4.8%), and neonatal death (1.2%) (Nayak 2022). This outcome is comparable to the one, where 2.8% of participants had CMV IgM antibodies.

### 4.4 Importance of the Study

We have highlighted TORCH infections and their impact. This information can enhance local healthcare practices by improving diagnostic capabilities and early screening protocols for pregnant women. Implementing routine TORCH screening in antenatal care can facilitate early detection, enabling timely interventions to prevent adverse pregnancy outcomes.

Furthermore, our study establishes a foundation for future research on public health interventions, including cost‐effective diagnostic methods, vaccination programs, and awareness campaigns. Longitudinal studies could also assess the effectiveness of these strategies in reducing the burden of TORCH infections and enhancing maternal and neonatal health outcomes.

### 4.5 Strengths of the Study

This study offers valuable insights into TORCH agent‐related infections in Pakistan. It serves as a foundation for the scientific community to further investigate this area and develop rapid diagnostic tests to prevent these infections.

### 4.6 Limitations of the Study

The sample size of 8000 is relatively small compared with the total population of the country, which is 225 million. Additionally, due to religious and privacy concerns, discussions regarding TORCH‐related infections have not been conducted openly.

## 5 Conclusion

Rubella infections are common among TORCH illnesses and are associated with higher rates of miscarriage compared with other infections. The prevalence of these illnesses has likely increased in Pakistan, primarily due to a lack of awareness, cultural restrictions, and a general reluctance among individuals to seek medical care during pregnancy. Our findings also indicate that TORCH infections adversely affect pregnancy and childbirth. Therefore, to reduce morbidity and mortality in our region, it is essential to understand the prevalence and impact of these infections during pregnancy. Implementing universal or targeted screening protocols for TORCH agents in pregnant women could facilitate early diagnosis and timely intervention, thereby reducing the risk of congenital infections and adverse pregnancy outcomes. Furthermore, integrating TORCH screening into national maternal health guidelines could raise awareness among healthcare providers and policymakers, ensuring standardized diagnostic practices. Future research should also evaluate the cost‐effectiveness and feasibility of such policies, ultimately improving maternal and neonatal health outcomes.

## Author Contributions

A.Z., A.A.E., and H.M.H. did the writing and manuscript supervision. L.S. did a formal analysis of the manuscript. R.M.E. did the conceptualization.

## Ethics Statement

The ASRB (Directorate of Advanced Studies & Research Board) of Quaid‐i‐Azam University, Islamabad.

## Consent

This form was waived by the Directorate of Advanced Studies & Research Board.

## Conflicts of Interest

The authors declare no conflicts of interest.

## Data Availability

All the data is mentioned and included in the manuscript.
